# Assessing the Association Between Meeting New 24‐h Movement Guidelines and Symptoms of Depression, Anxiety, and Stress Among Chinese Medical Students: A Multicenter Study

**DOI:** 10.1155/da/8848293

**Published:** 2025-12-04

**Authors:** Xinxin Ye, Zhuzhu Qin, Huanju Liu, Yining Tao, Wan Ye, Yanxia Zhong, Li Shu, Ruizhe Jiang, Cong Huang, Xinqiao Liu

**Affiliations:** ^1^ Department of Sports Science, Zhejiang University, Hangzhou, Zhejiang, China, zju.edu.cn; ^2^ Peoples Hospital of Deyang City, Deyang, Sichuan, China, dy120.net; ^3^ Department of Medical Genetics, Naval Medical University, Shanghai, China, smmu.edu.cn; ^4^ Clinical Research Center, Ningbo No. 2 Hospital, Ningbo, Zhejiang, China, nbws.gov.cn; ^5^ Department of Nursing, Xiamen Medical College, Xiamen, Fujian, China, xmmc.com.cn; ^6^ Department of Nursing, Shijiazhuang Medical College, Shijiazhuang, Hebei, China; ^7^ Department of Medicine and Science in Sports and Exercise, Tohoku University Graduate School of Medicine, Sendai, Miyagi, Japan, tohoku.ac.jp

## Abstract

**Objective:**

To investigate the associations between meeting the new Canadian 24‐h movement guidelines and symptoms of depression, anxiety, and stress among Chinese medical students, thus providing empirical and theoretical support for targeted mental health interventions.

**Methods:**

A total of 3679 medical students were recruited through multicenter convenience sampling in November 2022. A self‐administered standardized questionnaire assessed three 24‐h movement behaviors—moderate‐to‐vigorous physical activity (MVPA), sedentary time, and sleep—as well as symptoms of depression, anxiety, and stress. Logistic regression models were applied to analyze the association between meeting new 24‐h movement guidelines and symptoms of depression, anxiety, and stress.

**Results:**

A total of 3228 valid responses were obtained. The proportions of students who met all 24‐h movement behavior recommendations were low, at 4.01% among clinical students and 7.14% among nursing students. The overall prevalence of negative emotional symptoms was 60.9% in clinical students and 46.7% in nursing students. A clear dose–response relationship was evident between the number of recommendations met and a lower risk of depression, anxiety, and stress. Medical students who fully met all recommendations had a significantly lower risk of negative emotional symptoms compared with those who met none (stress odds ratio [OR] = 0.239, 95% confidence interval [CI]: 0.101–0.568, *p* = 0.001; anxiety OR = 0.601, 95% CI: 0.407–0.889, *p* = 0.011; depression OR = 0.450, 95% CI: 0.290–0.700, *p*  < 0.001). Similar protective associations were found in both clinical and nursing subgroups. All associations remained generally consistent after false discovery rate (FDR) correction, supporting the robustness of the results. A significant three‐way interaction among MVPA, sedentary behavior, and sleep was observed for stress.

**Conclusion:**

Adherence to the 24‐h movement behavior recommendations was suboptimal among Chinese medical students. However, greater adherence was associated with a lower risk of symptoms of depression, anxiety, and stress, highlighting the need for integrated lifestyle interventions targeting physical activity, sedentary time, and sleep balance.

## 1. Introduction

Globally, mental health issues among medical students are becoming increasingly severe. A systematic review and meta‐analysis covering 122,356 medical students from 43 countries, revealed that approximately 27.2% of them experienced depressive symptoms, and 11.1% had contemplated suicide [1]. In comparison, recent national data indicate that among Chinese medical students, the prevalence of depressive and anxiety symptoms is notably higher, at 57.5% and 30.8%, respectively [2]. These mental health problems severely impact not only the academic performance and career development of medical students but also the quality of future healthcare services. Given this alarming prevalence, identifying modifiable behavioral factors that contribute to symptoms of depression, anxiety, and stress among medical students has become a crucial global public health concern.

Growing attention has been directed toward the role of lifestyle behaviors in mental health in recent years. Insufficient sleep is widely recognized to be associated with increased negative emotional symptoms. Further supporting this association, a multicenter study involving medical students in Brazil revealed a significant correlation between sleep deprivation and increased symptoms of anxiety and depression [3]. Concurrently, screen exposure, particularly due to sedentary behavior and frequent use of electronic devices, has been confirmed to significantly increase the risk of anxiety and depression [4, 5]. Other studies have also found that increased screen time is closely associated with higher stress levels among medical students [6]. Moreover, a lack of physical activity is another critical factor affecting mental health. Compared with medical students who actively engage in moderate‐to‐vigorous physical activity (MVPA), those with insufficient activity levels are more likely to exhibit symptoms of anxiety and depression [7].

Although previous studies have separately revealed the associations between sleep duration, screen exposure, physical activity, and mental health, most of these studies have been limited to examining single dimensions. The concept of integrated 24‐h movement behaviors, which recognize the interconnection between sleep, sedentary behavior, and physical activity, have gained increasing attention in recent years [8]. The Canadian 24‐Hour Movement Guidelines for Adults, released in 2020 by the Canadian Society for Exercise Physiology, represent a significant advancement in public health promotion by advocating for a balanced integration of physical activity, sedentary behavior, and sleep within a 24‐h framework, which has demonstrated strong generalizability and has been extensively applied in both global and Chinese health research [9]. This guideline recommend that adults engage in at least 150 min of MVPA per week, limit sedentary time to no more than 8 h per day (including ≤3 h of recreational screen time), and obtain 7–9 h of good‐quality sleep per night. This framework reflects a paradigm shift from focusing on single behaviors to examining their combined impact on health and well‐being. However, most existing studies using the 24‐h movement framework have focused on adults with chronic diseases [10, 11], general university students [12–14], or health during the COVID‐19 pandemic [15, 16], while little is known about medical students—a population with unique academic stress and lifestyle constraints.

To our knowledge, this study is the first to apply the new Canadian 24‐h movement guidelines to Chinese medical students, a population that has not been previously examined within this integrated framework. Using multicenter data, we quantitatively evaluated the associations between 24‐h movement behaviors and mental health outcomes, including symptoms of depression, anxiety, and stress. In addition to assessing the independent effects of MVPA, sedentary behavior, and sleep, we further introduced interaction‐term analyses to explore potential synergistic or combined effects among these behaviors. This analytical approach extends the application of the 24‐h movement framework by capturing the interdependence between movement behaviors, which has been largely overlooked in prior single‐behavior studies. By extending the 24‐h movement paradigm to a culturally specific population facing rigorous academic and clinical training, this study provides novel empirical evidence and actionable insights for mental health promotion among future healthcare professionals.

## 2. Materials and Methods

### 2.1. Study Design and Participants

**Figure 1:**
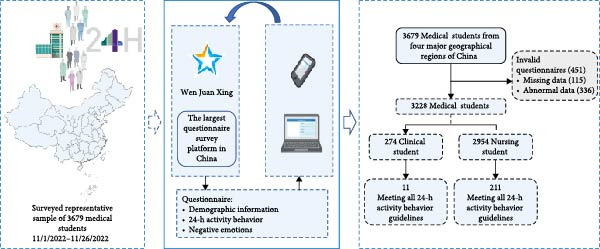
The study framework.

This is a multicenter cross‐sectional study. Participants completed the questionnaire via the online platform Wen juan xing (https://www.wjx.cn) from November 1, 2022 to November 26, 2022. The study employed a convenience sampling method. Medical students were recruited from three medical schools in China, spanning key geographical regions such as Hebei, Fujian, Anhui, Zhejiang, and Qinghai provinces. First, the project leader established a collaborative relationship with the administrators of the medical schools through communication. Subsequently, the project leader contacted the class advisors, who were responsible for the students’ daily academic and living affairs. The class advisors sent QR code posters or links to the students via WeChat or QQ, the two most commonly used social media platforms in mainland China, inviting them to complete the questionnaire [17]. Participants were informed that their participation in the study was voluntary, and their anonymous data would only be used for the research and would not be made public. A total of 3679 questionnaires were distributed, and 3228 valid responses were obtained after excluding incomplete or abnormal data, for a response rate of 87.7%. Details are shown in Figure 1.

Inclusion criteria: (1) medical students aged ≥18 years; (2) ability to independently read, comprehend, and complete the online questionnaire; (3) no known diagnosis of severe mental disorders (e.g., schizophrenia, bipolar disorder, or other psychotic conditions). All participants provided informed consent and voluntarily participated in the survey.

Exclusion criteria: (1) non–full‐time students, including those on medical or academic leave, those who had suspended their studies, or those enrolled in continuing education or other non–full‐time academic programs; (2) individuals unable to use a mobile phone or computer.

### 2.2. Sample Size Determination

The sample size was calculated using the single population proportion formula:

n=Zα/22×p 1−p/d2,

where *Z*_*α*/2_ = 1.96 for a 95% confidence level, *p* = 0.575 (estimated prevalence), and *d* = 0.05 (margin of error).

n=1.962×0.575376×10.575−/0.052=.

After adjusting for a 10% nonresponse rate and applying a design effect of 2 to account for the multicenter convenience sampling, the final required sample size was 828 participants. The achieved sample size of 3228 medical students exceeded this minimum requirement, ensuring adequate statistical power.

### 2.3. Questionnaire

The initial questionnaire was developed through a systematic literature review and a presurvey conducted with 10 students from different schools. Based on the presurvey results, the research team’s experts held discussions and made revisions, ultimately finalizing the questionnaire [8, 18]. The questionnaire consists of the following three main modules:

1. Demographic characteristics: age, gender, class level, whether they are an only child, ethnicity, average monthly income per capita, et cetera.
2. 24‐h Activity behavior.
 
  2.1. MVPA

Moderate‐intensity physical activity was assessed using the question from the International Physical Activity Questionnaire Short Form (IPAQ‐SF) [13]: “In the past 7 days, on how many days did you participate in moderate‐intensity physical activity (such as brisk walking, ballroom dancing, bowling, table tennis, badminton, cycling at a normal pace, or doubles tennis, etc.; please do not include walking) for more than 10 min?” and “On those days when you engaged in moderate‐intensity physical activity, how much time did you usually spend doing these activities?”

Vigorous‐intensity physical activity was assessed with the questions: “In the past 7 days, on how many days did you engage in vigorous physical activity (such as aerobic exercise, running, fast cycling, swimming, football, basketball, weightlifting, etc.) for more than 10 min?” and “On those days when you engaged in vigorous physical activity, how much time did you usually spend doing these activities?”

The total duration of MVPA over the past 7 days was calculated based on the number of days and the duration (hours and minutes) of each activity. The weekly total duration was then calculated. According to guidelines, 150 min of MVPA per week is recommended, and MVPA ≥150 min/week was defined as meeting the standard.

2.2. Sedentary Behavior

Sedentary behavior was assessed using the question from the IPAQ‐SF [19]: “In the past month, how much time did you spend sitting each day (including time spent sitting while studying or relaxing at home or school, as well as time spent sitting or lying while visiting friends, reading, or watching TV)?” This was used to calculate the total sedentary time. Additionally, participants were asked: “In the past month, how much time did you spend on your phone, TV, computer, or other electronic devices each day?” This was used to calculate the total screen exposure time. Guidelines recommend limiting daily sedentary behavior to <8 h/day, with screen exposure time <3 h/day. Meeting both of these recommendations was considered meeting the standard.

2.3. Sleep

Students reported their average daily sleep duration (including naps) over the past month. According to guidelines, the recommended sleep duration for adults is 7–9 h per night. The sleep duration compliance rate was calculated based on this recommendation.

3. Symptoms of depression, anxiety, and stress.

Symptoms of depression, anxiety, and stress in medical students were assessed using the 21‐item Depression Anxiety and Stress Scale (DASS‐21) [20]. Students filled out the scale based on their experiences in the past week, covering three dimensions: depression, anxiety, and stress. Each dimension includes seven items, with answers scored on a 0–3 scale, where 0 indicates “does not apply at all” and 3 indicates “applies very much.” The total score for each dimension was calculated by summing the individual item scores and multiplying by a factor of 2. A score of > 14 on the depression subscale, > 10 on the anxiety subscale, and > 19 on the stress subscale was considered indicative of the presence of corresponding symptoms of depression, anxiety, and stress. The Cronbach’s *α* coefficient for the DASS‐21 scale was 0.943, and for the subscales of depression, anxiety, and stress, the Cronbach’s *α* values ranged from 0.843 to 0.862. This scale has shown good reliability and validity when used among healthcare professionals.

### 2.4. Quality Control

This study adopted an online questionnaire format. All research personnel involved in the project received standardized training prior to the study’s commencement. Additionally, students were trained before completing the questionnaire. Each participant was assigned a unique questionnaire code to ensure individual responses. The questionnaire could only be submitted once it was fully completed. Additionally, real‐time monitoring of the data was conducted to ensure its reliability and accuracy.

### 2.5. Statistical Analysis

A database was constructed using Stata version 14.0 (Stata Corp LLC, College Station, TX, USA), excluding invalid or missing data prior to statistical analysis. Categorical variables were expressed as frequencies (%), and continuous variables were summarized as means ± standard deviations ($\overset{-}{x}$ ± s). Nonlinear associations between key variables were assessed using restricted cubic spline (RCS) logistic regression models, with the number and location of knots determined based on the minimum akaike information criterion (AIC). Analyses were conducted using the rcssci package in R. Results were reported as adjusted odds ratios (ORs) with 95% confidence intervals (CIs). Both overall and nonlinear *p*‐values were calculated to assess model fit and the statistical significance of nonlinear trends. After adjusting for confounding factors (age, gender, grade, and geographic origin), logistic regression analysis was performed to explore the relationship between 24‐h activity dimensions (MVPA, sedentary time, screen exposure time, and sleep) and symptoms of depression, anxiety, and stress. Additionally, logistic regression analysis was used to investigate the association between compliance with the recommended 24‐h movement guidelines (e.g., MVPA≥150 min/week, sedentary time <8 h/day, screen exposure time <3 h/day, and sleep 7–9 h/day) and emotional states (such as depression, anxiety, and stress). Given the multiple comparisons conducted—particularly in regression and subgroup analyses—false discovery rate (FDR) correction was applied using the Benjamini–Hochberg procedure to control for type I error inflation. A two‐tailed *p*‐value < 0.05 was considered statistically significant after correction. Finally, to assess potential synergistic or interactive effects among movement behaviors, interaction terms were included in the models (MVPA × sedentary behavior, MVPA × sleep, sedentary behavior × sleep, and MVPA × sedentary behavior × sleep).

## 3. Results

### 3.1. Demographic Characteristics of Participants by Major in Medical Students

A total of 3228 (87.7%) medical students were included in the final analysis. The mean age was 19.87 ± 1.27 years, with 2559 females (79.3%). Among the participants, 274 were clinical students (8.5%) and 2954 were nursing students (91.5%). The proportion of clinical students meeting all 24‐h activity behavior guidelines was 4.01%, while the proportion in nursing students was 7.14%. Conversely, the proportion of clinical students meeting none of the activity guidelines was 15.69%, while the proportion in nursing students was 17.37%. Clinical students had significantly shorter average sleep duration ([7.72 ± 1.22] h vs. [8.24 ± 1.42] h, *t* = −5.858, *p*  < 0.001) and less MVPA time (0.00 [0.00, 1.50] h vs. 0.00 [0.00, 3.00] h, *Z* = −2.049, *p* = 0.040) compared to nursing students. There were no significant differences between the two groups in terms of sedentary behavior or screen exposure time. The sleep duration compliance rate was significantly higher in clinical students than in nursing students (76.6% vs. 69.8%, *χ* ^2^ = 5.626, *p* = 0.018), whereas the compliance rates for MVPA (19.0% vs. 26.8%, *χ* ^2^ = 8.030, *p* = 0.005) and sedentary behavior (24.09% vs. 31.52%, *χ* ^2^ = 6.483, *p* = 0.011) were significantly lower in clinical students. Compared to nursing students, clinical students exhibited significantly higher levels of depression (17.8% vs. 23.7%, *χ* ^2^ = 5.868, *p* = 0.015) and stress (9.5% vs. 6.0%, *χ* ^2^ = 5.204, *p* = 0.023). See Table 1.

**Table 1:** Demographic characteristics of participants by major in medical students (*n* = 3228).

Characteristic and category	Total (*n* = 3228)	Clinical medical students (*n* = 274)	Nursing students (*n* = 2954)	Statistical value	*p*‐Value
Age (M [P25, P75])	19.87 ± 1.27	20.91 ± 1.96	19.78 ± 1.14	*t* = 14.52	<0.001
Gender, *n* (%)	—	—	—	*χ* ^2^ = 47.45	<0.001
Male	669 (20.72)	101 (36.86)	568 (19.23)	—	—
Female	2559 (79.28)	173 (63.14)	2386 (80.77)	—	—
Class level, *n* (%)	—	—	—	*χ* ^2^ = 610.88	<0.001
Freshman	2312 (71.62)	34 (12.41)	2278 (77.12)	—	—
Junior	799 (24.75)	182 (66.42)	617 (20.89)	—	—
Senior	117 (3.62)	58 (21.17)	59 (2.00)	—	—
Only child, *n* (%)	—	—	—	*χ* ^2^ = 7.79	0.005
Yes	516 (15.99)	60 (21.90)	456 (15.44)	—	—
No	2712 (84.01)	214 (78.10)	2498 (84.56)	—	—
Ethnicity, *n* (%)	—	—	—	*χ* ^2^ = 0.18	0.675
Han	3057 (94.70)	258 (94.16)	2799 (94.75)	—	—
Others	171 (5.30)	16 (5.84)	155 (5.25)	—	—
Place of origin, *n* (%)	—	—	—	*χ* ^2^ = 6.48	0.039
Urban	376 (11.65)	40 (14.60)	336 (11.37)	—	—
Town	608 (18.84)	62 (22.63)	546 (18.48)	—	—
Rural	2244 (69.52)	172 (62.77)	2072 (70.14)	—	—
Average monthly income per capita, CNY^a^, *n* (%)	—	—	—	*χ* ^2^ = 15.96	0.003
＜2000	718 (22.24)	67 (24.45)	651 (22.04)	—	—
2000–3000	981 (30.39)	63 (22.99)	918 (31.08)	—	—
3001–4000	577 (17.87)	47 (17.15)	530 (17.94)	—	—
4001–5000	368 (11.40)	27 (9.85)	341 (11.54)	—	—
＞5000	584 (18.09)	70 (25.55)	514 (17.40)	—	—
Living situation, *n* (%)	—	—	—	*χ* ^2^ = 132.404	<0.001
Alone	273 (8.46)	42 (15.33)	231 (7.82)	—	—
With family	1975 (61.18)	84 (30.66)	1891 (64.01)	—	—
With relatives	15 (0.46)	0 (0.0)	15 (0.51)	—	—
With friends	183 (5.67)	15 (5.47)	168 (5.69)	—	—
With classmates	744 (23.05)	122 (44.53)	622 (21.06)	—	—
Other	38 (1.18)	11 (4.01)	27 (0.91)	—	—
Recent graduate, *n* (%)	—	—	—	*χ* ^2^ = 41.80	<0.001
Yes	897 (27.79)	122 (44.53)	775 (26.24)	—	—
No	2331 (72.21)	152 (55.47)	2179 (73.76)	—	—
Sleep duration (hour, mean [SD])	8.20 ± 1.41	7.72 ± 1.22	8.24 ± 1.42	*t* = −5.86	<0.001
Sedentary time duration (hour, [M (P25, P75)])	4.00 (2.01, 6.00)	4.00 (2.50, 7.00)	4.00 (2.00, 6.00)	*Z* = −1.913	0.056
Screen exposure duration (hour, [M (P25, P75)])	3.00 (1.50, 5.21)	4.01 (2.00, 7.00)	3.00 (1.50, 5.02)	*Z* = 5.505	<0.001
MVPA duration (hour, [M (P25, P75)])	0.00 (0.00, 2.67)	0.00 (0.00, 1.50)	0.00 (0.00, 3.00)	*Z* = −2.049	0.040
Meeting sleep duration guideline, *n* (%)	2272 (70.4)	210 (76.6)	2062 (69.8)	*χ* ^2^ = 5.63	0.018
Meeting sedentary behavior guideline, *n* (%)	997 (30.89)	66 (24.09)	931 (31.52)	*χ* ^2^ = 6.48	0.011
Meeting MVPA guideline, *n* (%)	845 (26.2)	52 (19.0)	793 (26.8)	*χ* ^2^ = 8.03	0.005
Number of meeting criteria, *n* (%)	—	—	—	*χ* ^2^ = 9.387	0.025
0	556 (17.22)	43 (15.69)	513 (17.37)	—	—
1	1452 (44.98)	145 (52.92)	1307 (44.25)	—	—
2	998 (30.92)	75 (27.37)	923 (31.25)	—	—
3	222 (6.88)	11 (4.01)	211 (7.14)	—	—
Stress, *n* (%)	203 (6.3)	26 (9.5)	177 (6.0)	*χ* ^2^ = 5.20	0.023
Anxiety, *n* (%)	753 (23.3)	76 (27.7)	677 (22.9)	*χ* ^2^ = 3.26	0.071
Depression, *n* (%)	591 (18.3)	65 (23.7)	526 (17.8)	*χ* ^2^ = 5.87	0.015

Abbreviations: MVPA, moderate‐to‐vigorous physical activity; SD, standard deviation.

^a^Chinese Yuan.

### 3.2. Overall Association Between Adherence to Individual 24‐h Movement Guidelines and Symptoms of Depression, Anxiety, and Stress Among Medical Students

**Figure 2:**
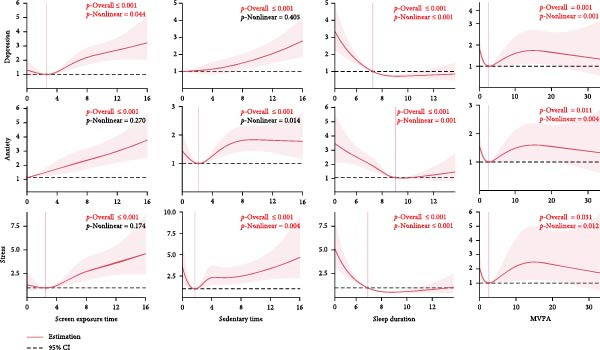
Nonlinear associations between adherence to individual 24‐h movement guidelines and symptoms of depression, anxiety, and stress. Results are derived from multivariable logistic regression models using restricted cubic spline (RCS) functions, adjusted for age, gender, grade level, only‐child status, place of origin, living situation, and graduation status. Solid lines represent adjusted odds ratios, and shaded areas indicate 95% confidence intervals. *p*‐overall and *p*‐nonlinear denote total and nonlinear significance, respectively.

To examine the nonlinear relationships between three psychological factors—depression, anxiety, and stress—and various physical activity parameters, including MVPA, sedentary time, screen exposure time, sleep, the RCS method was performed. As shown in Figure 2, increased screen exposure time and sedentary behavior were associated with progressively higher risks of depression, anxiety, and stress, while longer sleep duration demonstrated a U‐shaped relationship, with both insufficient and excessive sleep linked to higher odds of symptoms of depression, anxiety, and stress. MVPA exhibited an inverse association with psychological distress upto approximately 20 min per day, after which the curve plateaued. These findings highlight distinct threshold effects, suggesting that optimal balance across daily behaviors may be more important than any single activity dimension. Then, we adjusted the variables, such as age, gender, grade level, only‐child status, place of origin, living situation, and whether the student was a recent graduate, and the logistic regression analysis was conducted to find the independent factors of these psychological status. The results indicated that, among the overall population of medical students, meeting the guidelines for MVPA (OR = 0.763, 95% CI: 0.611–0.953), sedentary behavior (OR = 0.668, 95% CI: 0.542–0.824), and sleep (OR = 0.816, 95% CI: 0.672–0.992) were all associated with a lower risk of depression. Meeting the sedentary behavior guideline (OR = 0.672, 95% CI: 0.556–0.812) was associated with a lower risk of anxiety, but meeting the guidelines for MVPA and sleep showed no significant association with anxiety (*p* > 0.05). Meeting the sedentary behavior guideline (OR = 0.561, 95% CI: 0.393–0.801) and sleep guideline (OR = 0.712, 95% CI: 0.526–0.962) was significantly associated with lower risk of stress. Students meeting the sleep and screen exposure time guidelines had a significantly reduced risk of experiencing stress, although meeting the MVPA guideline was not significantly associated with stress (*p* > 0.05). After applying FDR correction to control for multiple comparisons, the associations between sleep and depression (FDR‐adjusted *p* = 0.265) and between sleep and stress (FDR‐adjusted *p* = 0.061) were no longer statistically significant, while other associations remained robust. Detailed results are available in the Table S1.

To examine potential interactive effects among movement behaviors, interaction terms (MVPA × sedentary behavior, MVPA × sleep, sedentary behavior × sleep, and MVPA × sedentary behavior × sleep) were included in the models. A significant three‐way interaction among MVPA, sedentary behavior, and sleep was observed for stress (OR = 0.135, 95% CI: 0.021–0.782, *p* = 0.028), indicating a lower stress risk when all three behaviors were jointly optimal. No significant interactions were found for depression or anxiety (*p* > 0.05). Detailed results are available in the Table S3.

### 3.3. Subgroup Analysis of the Effects of Adherence to Individual 24‐h Movement Guidelines on Symptoms of Depression, Anxiety, and Stress Among Medical Students by Majors

Among the nursing student population, the results showed that meeting the sedentary behavior guideline (OR = 0.710, 95% CI: 0.571–0.882, *p* = 0.002) was significantly associated with a lower risk of depression. Meeting the sedentary behavior guideline (OR = 0.706, 95% CI: 0.580–0.860, *p* = 0.001) was significantly associated with a lower risk of anxiety. Additionally, meeting the sedentary behavior guideline (OR = 0.630, 95% CI: 0.437–0.909, *p* = 0.013) was significantly associated with a reduced risk of stress, while meeting the MVPA (OR = 0.895, 95% CI: 0.622–1.286, *p* = 0.548) and sleep guidelines (OR = 0.756, 95% CI: 0.547–1.045, *p* = 0.090) showed no significant association with stress.

**Figure 3:**
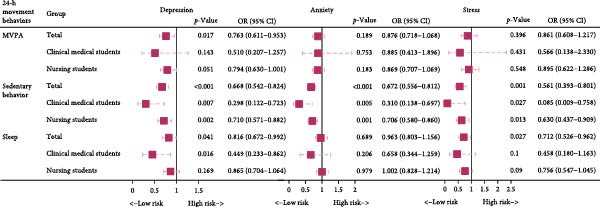
Association between adherence to individual 24‐h movement guidelines and symptoms of depression, anxiety, and stress among medical students. The model was adjusted for age, gender, grade level, only‐child status, place of origin, living situation, and whether the student was a recent graduate status. Additionally, the compliance statuses for sleep, sedentary behavior, and MVPA were mutually adjusted. The reference group was the noncompliant group for each type of activity. MVPA, moderate‐to‐vigorous physical activity.

In the clinical student population, meeting the sedentary behavior guideline was significantly associated with reduced risk of anxiety (OR = 0.310, 95% CI: 0.138–0.697, *p* = 0.005) and stress (OR = 0.085, 95% CI: 0.009–0.758, *p* = 0.027). Meeting the sedentary behavior (OR = 0.298, 95% CI: 0.122–0.723, *p* = 0.007) and sleep guidelines (OR = 0.449, 95% CI: 0.233–0.862, *p* = 0.016) were significantly associated with a lower risk of depression, although meeting the MVPA (OR = 0.510, 95% CI: 0.207–1.257, *p* = 0.143) showed no significant association with depression. See Figure 3. After applying FDR correction to account for multiple comparisons, the association between sedentary behavior and stress became nonsignificant (FDR‐adjusted *p* = 0.0607), whereas the relationships for anxiety and depression remained statistically robust. Detailed results are available in the Table S1.

### 3.4. Overall Association Between the Number of Met Components of the New 24‐h Movement Guidelines and Symptoms of Depression, Anxiety, and Stress in Medical Students

In the overall population of medical students, compared to meeting 0 components, meeting more components of the 24‐h movement guidelines was associated with a lower risk of symptoms of depression, anxiety, and stress. The risk of depression significantly decreased with the number of components met (meeting 1 component OR = 0.692, 95% CI: 0.546–0.876, *p* = 0.002; meeting 2 components OR = 0.530, 95% CI: 0.407–0.670, p  < 0.001; meeting 3 components OR = 0.450, 95% CI: 0.290–0.700, *p*  < 0.001). The risk of anxiety was significantly lower when meeting 2 or 3 components (meeting 2 components OR = 0.666, 95% CI: 0.520–0.852, *p* = 0.001; meeting 3 components OR = 0.601, 95% CI: 0.407–0.889, *p* = 0.011), while meeting 1 component was not significantly associated with anxiety risk (*p* = 0.112). The risk of stress decreased with an increase in the number of components met (meeting 1 component OR = 0.593, 95% CI: 0.413–0.849, *p* = 0.004; meeting 2 components OR = 0.524, 95% CI: 0.352–0.781, *p* = 0.002; meeting 3 components OR = 0.239, 95% CI: 0.101–0.568, *p* = 0.001).

### 3.5. Subgroup Analysis of the Effects of the Number of Met Components of the New 24‐h Movement Guidelines on Symptoms of Depression, Anxiety, and Stress Among Medical Students by Majors

Among nursing students, the risk of symptoms of depression, anxiety, and stress decreased as the number of components met increased. The risk of depression was significantly lower when meeting 1, 2, or 3 components (meeting 1 component OR = 0.715, 95% CI: 0.556–0.920, *p* = 0.009; meeting 2 components OR = 0.597, 95% CI: 0.453–0.788, *p*  < 0.001; meeting 3 components OR = 0.498, 95% CI: 0.316–0.784, *p* = 0.003). The risk of anxiety significantly decreased when meeting 2 or 3 components (meeting 2 components OR = 0.715, 95% CI: 0.552–0.926, *p* = 0.011; meeting 3 components OR = 0.631, 95% CI: 0.421–0.945, *p* = 0.025). For stress, this association was observed across all levels (meeting 1 component OR = 0.609, 95% CI: 0.411–0.901, *p* = 0.013; meeting 2 components OR = 0.608, 95% CI: 0.399–0.928, *p* = 0.021; meeting 3 components OR = 0.277, 95% CI: 0.115–0.667, *p* = 0.004).

**Figure 4:**
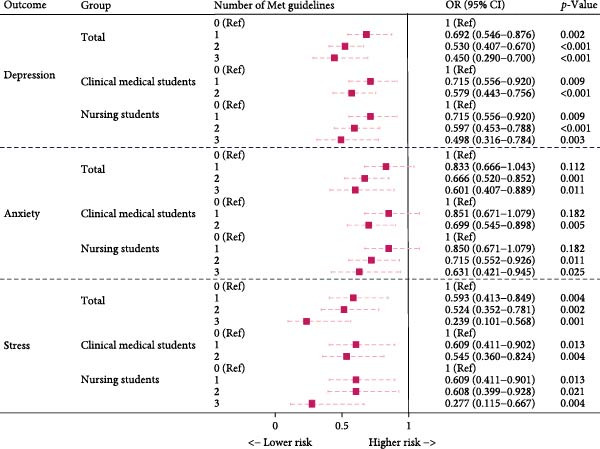
Association between the number of met components of the new 24‐h movement guidelines and symptoms of depression, anxiety, and stress among medical students. The model was adjusted for age, gender, grade level, only‐child status, place of origin, living situation, and whether the student was a recent graduate status. Among clinical medical students, due to the small sample size of individuals meeting all three components of the 24‐h movement activity guidelines (sleep, sedentary behavior, and MVPA), those meeting two and those meeting all three criteria were combined into a single category: meeting two or more criteria for the analysis. MVPA, moderate‐to‐vigorous physical activity.

Among clinical students, a similar trend was observed, with a lower risk of symptoms of depression, anxiety, and stress as the number of components met increased. The risk of depression was reduced with meeting 1, 2, or 3 components (meeting 1 component OR = 0.715, 95% CI: 0.556–0.920, *p* = 0.009; meeting 2 or more components OR = 0.579, 95% CI: 0.443–0.756, *p*  < 0.001). Anxiety risk was lower when meeting 2 or more components (OR = 0.699, 95% CI: 0.545–0.898, *p* = 0.005). The risk of stress decreased with meeting more components (meeting 1 component OR = 0.609, 95% CI: 0.411–0.902, *p* = 0.013; meeting 2 or more components OR = 0.545, 95% CI: 0.360–0.824, *p* = 0.004). Detailed results are available in Figure 4 and in the Table S2.

## 4. Discussion

This study analyzed the 24‐h movement patterns of Chinese medical students and their association with symptoms of depression, anxiety, and stress. The results showed that adherence to the 24‐h movement guidelines was generally insufficient among medical students, particularly among clinical students. Additionally, as the number of movement components met increased, the risk of symptoms of depression, anxiety, and stress significantly decreased, showing a dose–response effect. This gradient pattern reinforces the validity and practical relevance of the integrated 24‐h behavioral framework, enriching the existing evidence base of the 24‐h movement paradigm and extending its applicability to a culturally specific and high‐stress population—Chinese medical students. Together, these findings indicate that sleep, sedentary behavior, and physical activity are interdependent drivers of emotional health and that promoting adherence to Canada’s 24‐h movement guidelines may help prevent mental health problems in this population. Moreover, this discovery provides important scientific evidence for developing personalized health interventions tailored to medical students, contributing to the enhancement of both their mental and physical health and supporting sustainable career development.

The study found that MVPA was significantly associated with a lower risk of depression, consistent with extensive prior research indicating that regular physical activity can alleviate depressive symptoms. The mechanism involves increased secretion of brain neurotransmitters, such as endorphins, improved neuroplasticity, and enhanced immune function, all of which help improve mood [21]. However, the study did not find a significant association between MVPA and anxiety or stress, which may be due to the complex causes of these emotions, especially among medical students. These relationships are likely influenced by contextual factors, such as academic workload, personality traits, and social support. A study conducted at Helwan University found that approximately 93% of medical students experienced moderate‐to‐high levels of stress, with 54.9% experiencing moderate to concerning levels of anxiety. The primary stressors identified were academic, teaching, social, intrapersonal, and group activity‐related stressors, highlighting the significant impact of academic burdens on student well‐being [22]. Additionally, anxiety and stress may be closely linked to cognitive and emotional processing mechanisms, which physical activity alone may not fully alleviate [23, 24]. From a practical standpoint, this indicates that interventions targeting medical students should combine behavioral activation (e.g., physical activity) with psychological skill‐building, such as emotion regulation, mindfulness, or cognitive restructuring to achieve meaningful reductions in anxiety and stress symptoms. For clinical students specifically, although physical activity can help mitigate negative emotional symptoms, it may be insufficient to manage stress, anxiety, and depression when facing the high‐pressure internship environment. Therefore, future interventions should not only promote physical activity but also incorporate multilevel strategies that address emotional and psychological well‐being, such as time management, emotional regulation, and accessible mental health services [25, 26]. Specifically, universities and teaching hospitals may integrate structured physical activity into academic routines—such as group exercise or active breaks—while providing counseling, peer support, and stress management workshops to increase student engagement. This integrated approach could help strengthen resilience, improve coping under stress, and translate statistical associations into tangible psychological benefits in real‐life academic and clinical settings.

In this study, sedentary behavior was also found to be significantly associated with negative emotional symptoms, especially depression and anxiety. Research suggests that prolonged sedentary time, particularly the use of electronic devices, such as mobile phones and computers, can impair the brain’s emotional regulation functions [27]. This may be related to social isolation, cognitive overload, and sleep deprivation caused by excessive screen time, ultimately leading individuals to experience more emotional distress [28, 29]. This phenomenon is especially prevalent among medical students, particularly clinical students. Due to the high academic pressure, clinical students may rely more on screen devices for studying, resulting in higher screen exposure and, consequently, more depression symptoms [30]. Interestingly, among nursing students, screen exposure time was significantly associated with anxiety and depressive emotions but not with stress. This may reflect the dual nature of screen exposure in stress management. On one hand, prolonged study‐related screen exposure can exacerbate stress [31]; on the other hand, some medical students may use screen time as a way to relieve stress, such as by communicating with friends on social media or relaxing through entertainment, which might alleviate stress to some extent [32]. However, after applying the FDR correction, the association between sedentary behavior and stress among clinical medical students was no longer statistically significant, suggesting that this relationship may be less stable than that observed for depression and anxiety. This finding implies that sedentary behavior may influence emotional well‐being primarily through its impact on depressive and anxious symptoms rather than on stress per se. Furthermore, it is also possible that students experiencing higher emotional distress are more likely to engage in sedentary activities, highlighting the potential for reverse causality that warrants longitudinal verification. These findings suggest that not all sedentary activities carry the same psychological burden; differentiating between academic and recreational screen use could enhance the precision of behavioral recommendations and interventions. Therefore, screen time management should be individualized, with flexible intervention strategies based on professional field and study stage, combining healthy emotional coping methods like exercise and social interactions to balance screen use and mental health.

Insufficient sleep remains an important concern for mental health among medical students, particularly within high‐pressure clinical environments. Previous research has demonstrated that lack of sleep is closely associated with symptoms of depression, anxiety, and stress [33]. Mokros et al. [34] further demonstrated that poor sleep quality, assessed by the PSQI, independently predicts depressive symptoms in medical students, along with temperament and chronotype. In contrast to some previous findings, after FDR correction in the present study, sleep duration was not significantly associated with depression or stress in the overall medical student population. However, within the subgroup of clinical medical students, meeting the recommended sleep (7–9 h per night) remained significantly associated with a lower risk of depression. This suggests that adequate sleep may exert a stronger protective effect in individuals who face intensive clinical workloads and irregular schedules. The unique stressors experienced by clinical students—such as night shifts, heavy workloads, and emotional strain from patient care—may amplify the impact of insufficient sleep on mood regulation. Biologically, sleep deprivation may aggravate emotional disturbances through mechanisms involving hypothalamic–pituitary–adrenal (HPA) axis dysregulation [35] and inflammatory responses [36], which warrant further investigation in this high‐stress subgroup. By contrast, no significant association was found between sleep and anxiety, either in the overall population or within subgroups, suggesting that the causes of anxiety are complex and that improving sleep duration alone may not be sufficient to alleviate anxiety [37]. Anxiety among medical students often involves multiple interacting factors, such as uncertainty about future career choices and interpersonal stress, which may lie beyond the impact of sleep quality [38]. From a practical perspective, these findings indicate that sleep‐focused interventions may be particularly beneficial for clinical students, while broader psychosocial strategies are needed to address anxiety and stress in the general medical student population. Future mental health programs should, therefore, integrate sleep management with psychological counseling and stress reduction strategies to comprehensively improve emotional well‐being and resilience among medical students.

This study demonstrates that the risk of symptoms of depression, anxiety, and stress significantly decreases as the number of 24‐h movement guideline components met increases, further validating the importance of meeting multiple components. These components—physical activity, sedentary behavior, and sleep—are the key health behaviors outlined in the 24‐h movement guidelines, and collectively contribute to individuals’ physical and mental well‐being. In addition, the interaction analysis revealed a significant three‐way interaction among MVPA, sedentary behavior, and sleep for stress, suggesting that meeting all three recommendations simultaneously confers greater psychological benefits than meeting any single behavior alone. These results provide strong support for the “multi‐dimensional health behavior” concept emphasized in Canada’s 2020 24‐Hour Movement Guidelines for Adults [9]. The Canadian guidelines highlight that balancing physical activity, reducing sedentary time, and ensuring adequate sleep are key components for maintaining good mental health. By extending this integrated framework to a culturally specific and high‐stress population—Chinese medical students—this study enriches the existing evidence base and offers novel empirical support for the cross‐cultural applicability of the 24‐h movement paradigm. The findings suggest that the combined improvement of different components may be more effective in enhancing mental health than optimizing a single component [39]. Therefore, future interventions should focus on optimizing the overall 24‐h movement guideline components of medical students rather than improving individual components. Furthermore, developing tailored, culturally adapted health promotion strategies that integrate physical activity, sleep hygiene, and sedentary behavior management could provide a more comprehensive framework for enhancing both psychological well‐being and academic performance. Further research should also explore the specific needs of students in different majors to develop more targeted intervention plans that are applicable to both university and clinical settings, thereby effectively promoting the physical and mental health of medical students.

While this study successfully identified associations between 24‐h movement behaviors and symptoms of depression, anxiety, and stress among Chinese medical students, it does have some limitations. First, its cross‐sectional design precludes any inference of causality between 24‐h movement behaviors and symptoms of depression, anxiety, and stress, and longitudinal research is needed to clarify temporal relationships. Second, both movement behaviors and psychological outcomes were assessed through self‐reported questionnaires, which may introduce recall bias and common method variance. Third, the assessment of sedentary behavior and screen time was relatively limited, as it did not differentiate between academic screen exposure and recreational use, potentially leading to misclassification of sedentary patterns. In addition, the use of convenience sampling may have introduced recruitment bias, which restricts the representativeness of the sample and the generalizability of the findings. Finally, although the study primarily aimed to identify behavioral associations rather than to investigate underlying mechanisms, unmeasured psychosocial and physiological factors (e.g., academic stress, personality traits, and social support) may also influence both lifestyle behaviors and emotional outcomes, potentially confounding the observed associations. Future research should address these limitations by incorporating longitudinal or experimental designs to clarify causal relationships, and by using objective monitoring tools, such as accelerometers or wearable devices to more accurately capture 24‐h movement behaviors. Moreover, differentiating academic screen exposure from recreational use would allow a more precise understanding of how sedentary contexts contribute to mental health outcomes. Future studies are also encouraged to explore potential psychosocial and biological pathways linking movement behaviors with mental health to complement the population‐level evidence presented here.

## 5. Conclusion

Chinese medical students typically do not adhere sufficiently to the 24‐h movement guidelines. The risk of symptoms of depression, anxiety, and stress significantly decreases as more components of the guidelines are met. Furthermore, 24‐h movement behaviors among Chinese medical students are associated with different types of negative emotional symptoms. Specifically, MVPA and sleep are primarily related to depressive emotions, while meeting the sedentary behavior guidelines is closely associated with symptoms of depression, anxiety, and stress. A significant three‐way interaction among MVPA, sedentary behavior, and sleep was also observed for stress, indicating that integrating all three behaviors simultaneously may yield greater psychological benefits than focusing on any single behavior. Therefore, to improve the mental health of medical students more effectively, it is essential to rationally arrange their 24‐h movement behaviors and prioritize interventions targeting specific behaviors related to different negative emotional symptoms, including ensuring adequate sleep, limiting screen exposure time, and incorporating MVPA.

## Ethics Statement

The research involving human subjects received approval from the Medical Ethics Committee at Xiamen Medical College. All participants gave their written consent to participate in the study.

## Disclosure

All the authors have contributed to the article and approved the submitted version of this manuscript.

## Conflicts of Interest

The authors declare no conflicts of interest.

## Author Contributions

Xinxin Ye conceived, designed, carried out the study, data analysis, and prepared the first draft of the manuscript. Zhuzhu Qin and Huanju Liu helped design, data analysis, and prepared the manuscript. Yining Tao assisted in the data analysis and prepared the first draft of the manuscript. Wan Ye, Yanxia Zhong, Li Shu, and Ruizhe Jiang carried out the study and revise this manuscript. Cong Huang conceived, designed, and revise this manuscript. Xinxin Ye and Zhuzhu Qin contributed equally to this work.

## Funding

This study was funded by the Ministry of Education of Humanities and Social Science project, China (Grant 18YJCZH053), the Zhejiang Provincial Philosophy and Social Sciences Planning Project (Grant 24ZJQN054YB), and the National Social Science Fund of China (Grant 20FTYB014).

## Supporting Information

Additional supporting information can be found online in the Supporting Information section.

## Supporting information


**Supporting Information** Table S1. It presents the associations between adherence to individual 24‐h movement guidelines and symptoms of depression, anxiety, and stress among medical students, adjusted for multiple testing (FDR correction). Table S2. It presents the associations between the number of met components of the new 24‐h movement guidelines and symptoms of depression, anxiety, and stress in medical students, also adjusted for multiple testing (FDR correction). Table S3. It presents the associations between interaction effects of 24‐h movement behaviors and symptoms of depression, anxiety, and stress among medical students.

## Data Availability

The authors will provide the raw data underpinning the findings of this article to any qualified researcher upon reasonable request.
