# Supplementary material for: Assessing the Association Between Meeting New 24‐h Movement Guidelines and Symptoms of Depression, Anxiety, and Stress Among Chinese Medical Students: A Multicenter Study
Source: Depress Anxiety. 2025 Dec 4;2025:8848293. doi: 10.1155/da/8848293 (PMC12698267; doi:10.1155/da/8848293)
Supplement: Supplementary file 1 — Supporting Information Table S1. It presents the associations between adherence to individual 24‐h movement guidelines and symptoms of depression, anxiety, and stress among medical students, adjusted for multiple testing (FDR correction). Table S2. It presents the associations between the number of met components of the new 24‐h movement guidelines and symptoms of depression, anxiety, and stress in medical students, also adjusted for multiple testing (FDR correction). Table S3. It presents the associations between interaction effects of 24‐h movement behaviors and symptoms of depression, anxiety, and stress among medical students. [file DA-2025-8848293-s001.docx]

**Supporting Information**

**Table S1** **Association Between Adherence to Individual 24-Hour Movement Guidelines and Symptoms of Depression, Anxiety, and Stress Among Medical Students, Adjusted for Multiple Testing (FDR Correction)**

| Compliance items | | Total | | | Clinical medical students | | | Nursing students | | |
| --- | --- | --- | --- | --- | --- | --- | --- | --- | --- | --- |
|  |  | **95%*CI*** | ***Statistic P value*** | | **95%*CI*** | ***Statistic P value*** | | **95%*CI*** | ***Statistic P value*** | |
| Stress | **MVPA** | 0.861(0.608-1.217) | p_value | 0.396 | 0.566(0.138-2.330) | p_value | 0.431 | 0.895(0.622-1.286) | p_value | 0.548 |
|  |  |  | global_fdr | 0.486 |  | global_fdr | 0.506 |  | global_fdr | 0.6165 |
|  | **Sedentary Behavior** | 0.561(0.393-0.801) | p_value | 0.001 | 0.085(0.009,0.758) | p_value | 0.027 | 0.630(0.437-0.909) | p_value | 0.013 |
|  |  |  | global_fdr | 0.0067† |  | global_fdr | 0.0607 |  | global_fdr | 0.0439† |
|  | **Sleep** | 0.712(0.526-0.962) | p_value | 0.027 | 0.458(0.180-1.163) | p_value | 0.1 | 0.756(0.547,1.045) | p_value | 0.09 |
|  |  |  | global_fdr | 0.061 |  | global_fdr | 0.1688 |  | global_fdr | 0.162 |
| Anxiety | **MVPA** | 0.876(0.718-1.068) | p_value | 0.017 | 0.885(0.413-1.896) | p_value | 0.143 | 0.869(0.707-1.069) | p_value | 0.051 |
|  |  |  | global_fdr | 0.0459† |  | global_fdr | 0.2271 |  | global_fdr | 0.0984 |
|  | **Sedentary Behavior** | 0.672(0.556-0.812) | p_value | <0.001 | 0.310(0.138-0.697) | p_value | 0.007 | 0.706(0.580-0.860) | p_value | 0.002 |
|  |  |  | global_fdr | 0.0022† |  | global_fdr | 0.0270† |  | global_fdr | 0.0108† |
|  | **Sleep** | 0.963(0.803-1.156) | p_value | 0.041 | 0.658(0.344-1.259) | p_value | 0.016 | 1.002(0.828-1.214) | p_value | 0.169 |
|  |  |  | global_fdr | 0.085 |  | global_fdr | 0.0459† |  | global_fdr | 0.2535 |
| Depression | **MVPA** | 0.763(0.611-0.953) | p_value | 0.189 | 0.510(0.207-1.257) | p_value | 0.753 | 0.794(0.630-1.001) | p_value | 0.183 |
|  |  |  | global_fdr | 0.255 |  | global_fdr | 0.782 |  | global_fdr | 0.2551 |
|  | **Sedentary Behavior** | 0.668(0.542-0.824) | p_value | <0.001 | 0.298(0.122-0.723) | p_value | 0.001 | 0.710(0.571-0.882) | p_value | 0.005 |
|  |  |  | global_fdr | 0.0010† |  | global_fdr | 0.0067† |  | global_fdr | 0.0225† |
|  | **Sleep** | 0.816(0.672-0.992) | p_value | 0.206 | 0.449(0.233-0.862) | p_value | 0.689 | 0.865(0.704-1.064) | p_value | 0.979 |
|  |  |  | global_fdr | 0.265 |  | global_fdr | 0.7441 |  | global_fdr | 0.979 |

Notes: OR=Odds Ratio; MVPA=Moderate to Vigorous Physical Activity.

† Global FDR<0.05: Global correction (correcting all 27 tests). FDR correction using the Benjamini Hochberg method

**Table S2 Association Between the Number of Met Components of the New 24-Hour Movement Guidelines and Symptoms of Depression, Anxiety, and Stress in Medical Students, Adjusted for Multiple Testing (FDR Correction)**

| Dependent variable | Number of met  24-hour activity guidelines | Total | | | Clinical medical students | | | Nursing students | | |
| --- | --- | --- | --- | --- | --- | --- | --- | --- | --- | --- |
|  |  | **95%CI** | ***Statistic P value*** | | **95%CI** | ***Statistic P value*** | | **95%CI** | ***Statistic P value*** | |
| Stress | **0** | Ref |  |  | Ref |  |  | Ref |  |  |
|  | **1** | 0.593(0.413-0.849) | p_value | 0.004 | 0.609(0.411-0.902) | p_value | 0.013 | 0.609(0.411-0.901) | p_value | 0.013 |
|  |  |  | global_fdr | 0.0080† |  | global_fdr | 0.0164† |  | global_fdr | 0.0164† |
|  | **2** | 0.524(0.352-0.781) | p_value | 0.002 | 0.545(0.360-0.824) | p_value | 0.004 | 0.608(0.399-0.928) | p_value | 0.021 |
|  |  |  | global_fdr | 0.0060† |  | global_fdr | 0.0080† |  | global_fdr | 0.0252† |
|  | **3** | 0.239(0.101-0.568) | p_value | 0.001 | —— | p_value | —— | 0.277(0.115-0.667) | p_value | 0.004 |
|  |  |  | global_fdr | 0.0040† |  | global_fdr |  |  | global_fdr | 0.0080† |
| Anxiety | **0** | Ref |  |  | Ref |  |  | Ref |  |  |
|  | **1** | 0.833(0.666-1.043) | p_value | 0.112 | 0.851(0.671-1.079) | p_value | 0.182 | 0.850(0.671-1.079) | p_value | 0.182 |
|  |  |  | global_fdr | 0.1222 |  | global_fdr | 0.182 |  | global_fdr | 0.182 |
|  | **2** | 0.666(0.520-0.852) | p_value | 0.001 | 0.699(0.545-0.898) | p_value | 0.005 | 0.715(0.552-0.926) | p_value | 0.011 |
|  |  |  | global_fdr | 0.0040† |  | global_fdr | 0.0092† |  | global_fdr | 0.0155† |
|  | **3** | 0.601(0.407-0.889) | p_value | 0.011 | —— | p_value | —— | 0.631(0.421-0.945) | p_value | 0.025 |
|  |  |  | global_fdr | 0.0155† |  | global_fdr |  |  | global_fdr | 0.0286† |
| Depression | **0** | Ref |  |  | Ref |  |  | Ref |  |  |
|  | **1** | 0.692( 0.546-0.876) | p_value | 0.002 | 0.715(0.556-0.920) | p_value | 0.009 | 0.715(0.556-0.920) | p_value | 0.009 |
|  |  |  | global_fdr | 0.0060† |  | global_fdr | 0.0144† |  | global_fdr | 0.0144† |
|  | **2** | 0.530(0.407-0.670) | p_value | <0.001 | 0.579(0.443-0.756) | p_value | <0.001 | 0.597(0.453-0.788) | p_value | <0.001 |
|  |  |  | global_fdr | <0.001† |  | global_fdr | 0.0021† |  | global_fdr | 0.0021† |
|  | **3** | 0.450(0.290-0.700) | p_value | <0.001 | —— | p_value | —— | 0.498(0.316-0.784) | p_value | 0.003 |
|  |  |  | global_fdr | 0.0023† |  | global_fdr |  |  | global_fdr | 0.0080† |

Notes: OR=Odds Ratio; MVPA=Moderate to Vigorous Physical Activity.

† Global FDR<0.05: Global correction (correcting all 27 tests). FDR correction using the Benjamini Hochberg method

**Table S3 Association Between Interaction Effects of 24-Hour Movement Behaviors and Symptoms of Depression, Anxiety, and Stress Among Medical Students**

| **Dependent**  **variable** | **Compliance items** | **Total** | | | **Clinical medical students** | | | **Nursing students** | | |
| --- | --- | --- | --- | --- | --- | --- | --- | --- | --- | --- |
|  |  | **OR (95%*CI)*** | ***P* value** | ***AIC*** | **OR (95%*CI)*** | ***P* value** | ***AIC*** | **OR (95%*CI)*** | ***P* value** | ***AIC*** |
| **Stress** | **MVPA*Sedentary Behavior** | 1.066 (0.472, 2.311) | 0.874 | 1507.091 | —— | —— | —— | —— | —— | —— |
|  | **MVPA*Sleep** | 0.935 (0.469, 1.900) | 0.850 | 1513.398 | 0.346 (0.012, 5.443) | 0.460 | 179.6865 | 0.346 (0.012, 5.443) | 0.460 | 173.7238 |
|  | **Sleep*Sedentary Behavior** | 1.455 (0.669, 3.393) | 0.361 | 1502.228 | —— | —— | —— | —— | —— | —— |
|  | **MVPA*Sedentary Behavior*Sleep** | 0.135 (0.021, 0.782) | 0.028 | 1504.396 | —— | —— | —— | —— | —— | —— |
| **Anxiety** | **MVPA*Sedentary Behavior** | 1.031 (0.672, 1.570) | 0.888 | 3461.662 | 0.761 (0.086, 4.849) | 0.784 | 327.6862 | 0.761 (0.086, 4.849) | 0.789 | 327.6862 |
|  | **MVPA*Sleep** | 1.391(0.906, 2.160) | 0.136 | 3476.847 | 1.108 (0.204, 6.963) | 0.907 | 335.6212 | 1.108 (0.204, 6.963) | 0.907 | 335.6212 |
|  | **Sleep*Sedentary Behavior** | 1.195 (0.776,1.866) | 0.425 | 3462.619 | 2.471 (0.394, 21.637) | 0.360 | 325.3939 | 2.471 (0.394, 21.637) | 0.360 | 325.3939 |
|  | **MVPA*Sedentary Behavior*Sleep** | 1.261 (0.464,3.560) | 0.654 | 3466.859 | —— | —— | —— | —— | —— | —— |
| **Depression** | **MVPA*Sedentary Behavior** | 1.142 (0.705, 1.831) | 0.584 | 3045.400 | 0.822 (0.036, 7.408) | 0.875 | 309.2605 | 0.601 (0.093, 4.208) | 0.603 | 309.2605 |
|  | **MVPA*Sleep** | 1.503 (0.937, 2,448) | 0.096 | 3053.588 | 0.610 (0.093, 4.208) | 0.603 | 312.0043 | 0.822 (0.036, 7.408) | 0.875 | 312.0043 |
|  | **Sleep*Sedentary Behavior** | 1.213 (0.762, 1.960) | 0.423 | 3046.763 | 2.526 (0.374, 22.916) | 0.361 | 305.1005 | 2.526 (0.374, 22.916) | 0.361 | 305.1005 |
|  | **MVPA*Sedentary Behavior*Sleep** | 0.928 (0.313, 2.848) | 0.893 | 3046.434 | —— | —— | —— | —— | —— | —— |

Notes: MVPA=Moderate to Vigorous Physical Activity. The model was adjusted for age, gender, grade level, only-child status, place of origin, living situation, and whether the student was a recent graduate status. Additionally, the compliance statuses for sleep duration, sedentary behavior, and MVPA were mutually adjusted. The reference group was the non-compliant group for each type of activity. In subgroup analyses, some interaction estimates could not be reliably calculated due to small sample sizes in certain combinations of physical activity, sleep, and sedentary behavior, resulting in missing values.
